# Spinal cord injury modeling: from modeling to evaluation using rats as examples

**DOI:** 10.3389/fneur.2025.1573779

**Published:** 2025-06-16

**Authors:** Shuai Wang, Suying Cai, Zhemin Zhu, Weibo Zeng, Shengxuan Hu, Benchao Shi

**Affiliations:** ^1^Department of Spinal Surgery, Orthopedic Medical Center, Zhujiang Hospital, Southern Medical University, Guangzhou, China; ^2^Department of Pediatric Orthopedics, Dongguan Maternal and Child Health Hospital, Dongguan, China; ^3^Department of Spine Surgery, The Second Xiangya Hospital, Central South University, Changsha, China; ^4^Department of Orthopedic, Affliated Hospital of Jiujiang University, Jiujiang, China

**Keywords:** spinal cord injury, animal models, contusion models, behavioral assessment, pathophysiological processes

## Abstract

Spinal cord injury (SCI), with its enormous impact on individuals and society, seriously affects patients’ quality of life and is the focus and challenge of current medical research. The selection of appropriate SCI models and the reduction of heterogeneity between models are crucial for basic research on SCI. Although many articles have summarized and compared various SCI models, there are limited descriptions of how to further select the model animals after selecting the type of model, the degree of SCI, the use of anesthesia and analgesia, experience with modeling techniques, preoperative and postoperative care, management of common complications, sample collection, and evaluation of the spinal cord after injury. This paper aims to provide a practical guide for researchers who need to construct SCI models by combining the experimental experience of our research team in modeling and other related research literature. These guidelines will promote the standardization of SCI models, thus providing a solid foundation for in-depth research on SCI and the development of therapeutic strategies.

## Highlights

- Compression SCI is more accessible to generalize and is compatible with the clinical pattern of SCI.
- Rodents are the most cost-effective animals for SCI modeling, and rats are more consistent with the pathophysiological pattern of human SCI than mice.
- Preoperative fasting is not required for SCI modeling.
- SCI model animals can be treated adequately with analgesics without behavioral effects.
- Animals following spinal cord injury modeling that meet the criteria for euthanasia must be euthanized promptly for humanitarian endpoints, and it is not ethical or scientifically sound to force this animal to remain and be included in the experimental data.
- Treatment of postoperative complications after SCI.
- Tips for collecting animal specimens from SCI models.

## 1 Introduction

According to the World Health Organization (WHO), the number of new SCI patients worldwide ranges from 250,000 to 500,000 per year, and with the popularization of automobiles and other means of transportation and the aging of society, the number of spinal cord injuries due to car accidents, fall, and other accidents has been increasing every year (1). Functional recovery after SCI is challenging, which not only poses a significant threat to the physical and mental health of patients but also imposes a heavy economic burden on families and society (2). It is estimated that more than 27 million people worldwide are currently chronically disabled due to SCI (3). Because of this, SCI repair research has become particularly critical and is a challenging and hot spot in current scientific research. An accurate and appropriate animal model in SCI research is crucial for simulating human SCI and testing treatments (4). This review does not primarily aim to delineate the distinctions between different SCI models, but rather seeks to provide practical guidance for researchers engaged in SCI model establishment. It focuses on summarizing common challenges encountered during model construction and offers potential solutions based on experimental experience. Key topics addressed include the selection of animal species and sex, the choice of appropriate injury model types, technical considerations in surgical procedures, management of post-injury complications, methodologies for spinal cord and blood sample collection, as well as comprehensive strategies for post-injury evaluation. This paper aims to summarize the essential techniques and evaluation indices of SCI animal models from the perspective of SCI modeling to provide a reference for future research.

## 2 Current state of SCI modeling

There is a wide variety of spinal cord injury models, and the advantages and disadvantages of the models and their application areas are summarized in Table 1. They can be divided into contusion injury, transection injury, compression injury, chemical injury, traction subluxation injury, etc. (5–7); they can also be divided into acute SCI models and chronic SCI models (8). There are many SCI injury models, and how to choose a suitable one for oneself is none other than the following (9): 1. Controllability: the degree of injury must be controllable to suit different research purposes; 2. Reproducibility: the model can be replicated by others with high credibility and substantial implementability. 3. Clinical similarity: SCI models should be clinically relevant to patients’ injuries. 4. Operationalization: the model should be made objective and reasonable, and the instruments used should be simplified as much as possible.

**Table 1:** Summary of SCI models.

Type of injury	Use of tools	Vantage	Drawbacks	Scope of application	References	Year developed
Contusion model	NYU/ MASCIS Impactor	It is widely used and produces validated and reproducible contusions in rat models.	Bouncing of the impact bar may result in multiple impacts, affecting the consistency of the damage.	The contusion model is more consistent with the clinical spinal cord injury pattern and is suitable for studying the pathophysiological changes, repair, and regeneration patterns after SCI.	(42, 89–92)	1992
	IH Impactor	Avoid secondary injuries by applying damage through force control rather than weight drop.	Variability in clamping the spine may affect the consistency of the injury.		(35, 93, 94)	2003
	OSU Impactor	Measurement of force applied to the spinal cord and spinal cord displacement.	Challenges may be faced in determining the zero distance of the impactor.		(95)	1987
	Air-Gun Impactor	Minimally invasive, no need to cut through the vertebral plate to avoid accidental damage to the spinal cord	Lack of precise injury quantification requires further validation of model reliability and repeatability.		(96)	2012
Transverse injury modeling	Surgical blades or scissors or needles (complete transection)	Produces consistent and reliable cut-off injuries that are easy to enforce.	Complete severance of the spinal cord is less common in clinical situations.	It is suitable for studying axonal regeneration and degeneration.	(97)	–
	Surgical blades or scissors or needles (partial transection)	Complete severance of the spinal cord is less common in clinical situations.	Applying consistent injuries is challenging, and it may be difficult to determine if the targeted nerve bundle is completely severed.		(98, 99)	–
Crush injury	Aneurysm Clip/Artery Clip	These models simulate compression-extrusion spinal cord injuries, offering a cost-effective and straightforward approach without the need for complex equipment. These models align relatively well with the typical patterns observed in clinical spinal cord injuries.	Difficulty in precisely controlling the force of the impact and the degree of compression of the spinal cord.	Suitable for studies related to compression injury to the spinal cord.	(100)	1978
	Tweezers		Lack of simulation of acute injury and consistency of injury may be compromised.		(101)	1991
	Bolts			To simulate the effects of lumbar disc herniation and slipped discs on the spinal cord and to study studies related to spinal cord injury in this model.	(102)	2008
	Balloon				(103)	1954
	Spinal cord strapping	No disruption of the dura mater, reducing modeling variability and adverse effects associated with surgery	Molding is technically complex, and damage consistency needs to be further verified.		(104)	2008
Photochemical damage modeling		No removal of the lamina is required, and the dura is intact	The damage’s location, extent, and nature are difficult to control accurately and have poor repeatability.	Correlative studies examining secondary injury to the spinal cord following trauma	(105)	1987
Pulling dislocation model		Good simulation of clinical spinal cord injury due to pulling subluxation	Complex equipment setup that requires further validation and evaluation.	A correlative study of spinal cord injuries caused by traction subluxation	(5, 106)	2004
Ischemia model		Without the removal of the vertebrae, the dura mater is intact. Easy to make and reproducible.	This can easily lead to other organ damage.	Research related to the study of spinal cord ischemia, mechanisms related to spinal cord ischemia and reperfusion, and therapy	(107)	1987
Excitotoxicity model		There is no need to remove the vertebrae, as the dura is intact.	Poor clinical similarity, not easy to control the degree of damage	Research SCI Pathophysiology	(108)	1993
Electrical damage modeling		There is no need to remove the vertebrae.	Poor clinical similarity, not easy to control the degree of damage	Studying neuronal pathways in the central nervous system.	(109)	2008

Full English names of abbreviations used in the table. SCI, spinal cord injury; NYU, New York University; MASCIS, multicenter animal spinal injury study; OSU, Oregon State University; IH, infinite horizon.

Regarding the progress of SCI model, most studies adopt the contusion model (7). Most of the instruments used in the contusion model are commercialized or homemade professional strikers with complex structures and operations, which are expensive and difficult to move (6). The homemade strikers, although simple in construction, are unable to control the problem of varying degrees of injury caused by the rebound of the striking column after SCI.

The transection injury model ranked second in terms of utilization; this injury pattern occurs rarely in clinical patients and does not align with clinical similarities (10). In addition to complete transection, the remaining left and right semi-transection or ventral-dorsal transection or specific injury to a particular nerve bundle are relatively uncontrollable, and it is challenging to ensure that the degree of injury is the same in each animal.

Today’s main tools used to model compression SCI are forceps and special clips (11). The clips used for SCI modeling are mainly aneurysm clips or ordinary vascular clips, the former being the most common (7). The aneurysm clips and clamps used in most current articles are sophisticated and expensive, and the aneurysm clips have fatigue properties of the metal after repeated opening and closing, resulting in a gradual reduction of the clamping force from the original calibrated pressure (12, 13). The aneurysm clips must be replaced after a certain number of openings and closings, and the timing of replacement is problematic and adds significantly to the cost of the model.

Some SCI models use forceps closure to model SCI, which is a simple method that simulates the familiar site of SCI in humans-ventral injuries-without destroying the dura mater but has slightly poorer reproducibility of modeling (4). Current research has resulted in many clamp-type SCI modeling methods with modifications to the forceps (10). The size of the opening and closing of the forceps is controlled by adding spacers, limiters, etc. in the center of the forceps (14). In some cases, the legs of the forceps have been modified to be parallel to create an SCI model with smaller differences. However, accessing these modified modeling tools is difficult for the average researcher (4). The pathophysiological changes in SCI after SCI are very complex and vary from SCI model to SCI model (4, 15). The selection of SCI animal models must be reproducible and manipulable to achieve a high degree of homogeneity.

## 3 Modeling of spinal cord injuries

### 3.1 Selection of SCI animals

The most commonly used animal models for SCI are rodents, of which rats are the most commonly used at 72.4% (7). The others, in descending order of use, are mice, rabbits, dogs, and cats (7, 16). However, large animals and non-human primates such as pigs and rhesus monkeys are still the closest to human SCI (17–20). However, they are generally infrequently used due to strict regulatory and ethical requirements and practical economics (21).

#### 3.1.1 Rats

**Figure 1:**
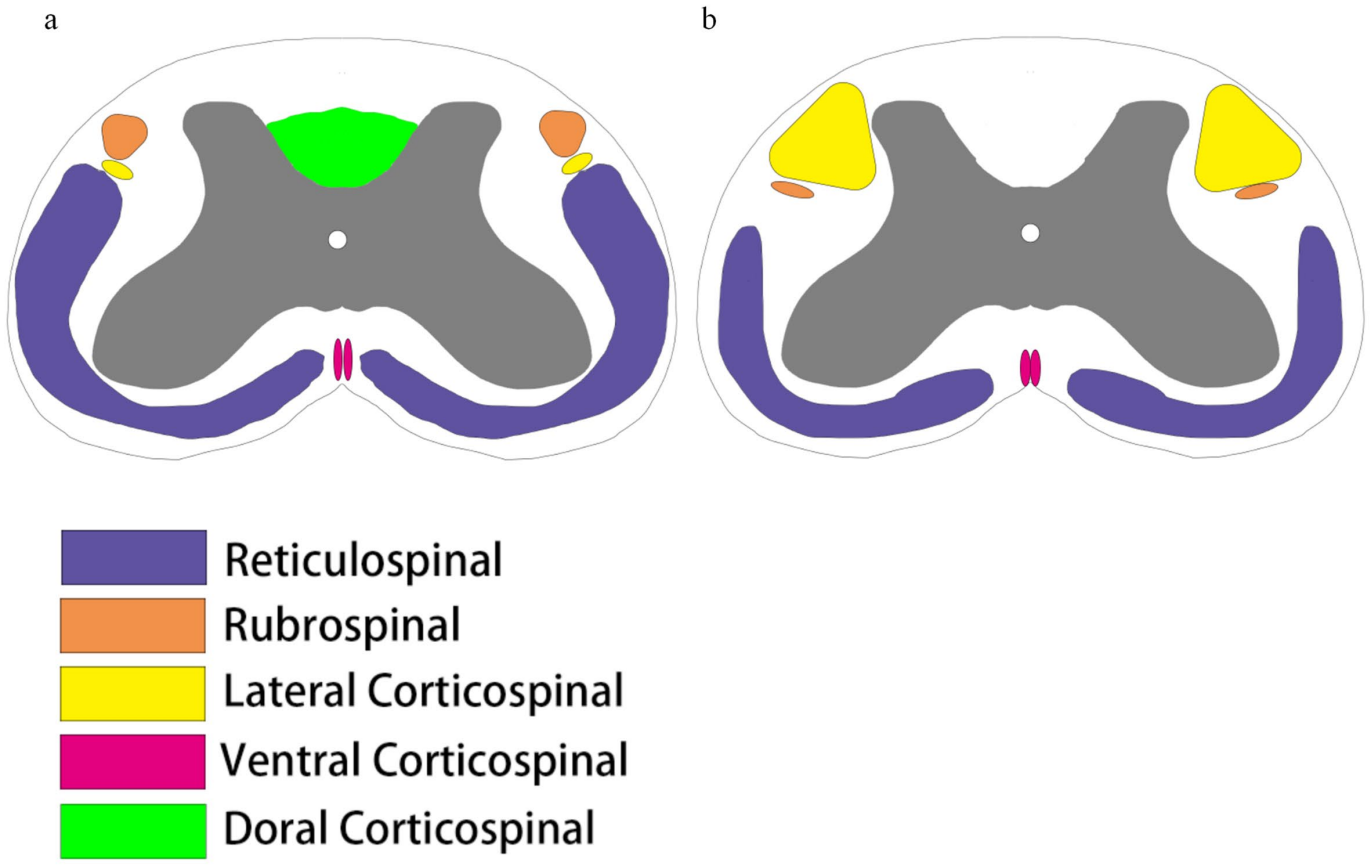
Comparison of spinal cord bundles. **(a)** The spinal cord tracts running down the rat spinal cord include the reticular spinal cord tracts (purple), the red nucleus pulposus tracts (brown), and the corticospinal tracts, which are divided into the corticospinal tracts of the lateral cortical spinal cord (yellow), the ventral corticospinal tracts (peach), and the dorsal corticospinal tracts (fluorescent green). **(b)** Spinal cord tracts running down the human spinal cord include the reticular spinal cord tracts (purple), the red nucleus spinal cord tracts (brown), and the corticospinal tracts, which are divided into the lateral corticospinal tracts (yellow) and the ventral corticospinal tracts (peach). Humans do not have dorsal corticospinal tracts, and there are some differences in the location and size of the distribution of the tracts in rats and humans.

In SCI research, rats are widely used as experimental models because of their unique biological characteristics. The following are the main advantages of rats as SCI models and key points to note: First, the pathological changes after SCI are similar to those in humans and can form similar special pathological manifestations such as scarring, cavities, and fluid-filled cysts (16, 22). Second, it is highly resistant to infection and is not prone to death due to surgical infection or postoperative urinary retention infection, and the survival rate of SCI models is high and relatively inexpensive (23). Because of its body and moderate size, it is relatively easy to perform SCI surgery without the need for delicate microscopic manipulation as in mice, and avoids the complex modeling process as in large animals such as rabbits or pigs, making the rat a cost-effective model. However, it should be noted that rats are rodents, and their corticospinal tracts are mainly dorsal, and the individual fiber tracts are somewhat different from those of humans; if SCI modeling requires damage to specific fiber tracts, it must be considered in conjunction with cross-sectional mapping of the rat (24–27) (Figure 1). Another point that requires special attention is that the thoracic spine of rats consists of 13 vertebrae, and special attention must be paid to localization during SCI modeling (28).

#### 3.1.2 Mice

Mice are essential in studying pathophysiological mechanisms and repair of SCI due to their ease of genetic modification (29). Through gene knockout or high expression techniques, researchers can probe the molecular basis of SCI more deeply. However, the relatively complex process of genetic modification in rats compared to mice has limited its application in certain genetic studies (30). Mice are more suitable for genetic manipulation and mechanistic studies due to well-established transgenic lines and genome editing techniques, while rats are preferred in surgical models owing to their size and surgical accessibility. The model choice should be based on the study focus—mechanistic exploration versus surgical intervention. Notably, the pattern of SCI recovery in mice differs significantly from that of human recovery after injury. Mice are highly self-recovery, and the proliferation of cells at the lesion site may lead to moderate regeneration even after complete transection, whereas in rats and humans, wholly transected SCIs generally do not recover (26). The histopathology of SCI in mice differs from that in humans, mainly in the time point of inflammatory cell infiltration and the difference in the composition of the tissue in the core area of the lesion in chronic SCI (31).

#### 3.1.3 Animal sex selection

Spinal cord injury (SCI) research relies heavily on animal models to understand pathogenesis and test potential treatments. The choice of animal sex in these models is critical because biological differences between males and females can affect the outcome of SCI studies (32). In clinical practice most SCI cases occur in males, but female animals are chosen to model most SCI models (33). The probable reason for this is that female animals have stronger anti-inflammatory levels and are able to achieve higher survival rates after SCI (34). Female animals have a wider urethra, which makes it easier to assist urination, and urinary retention is relatively less painful (35). There is some protective effect of estrogen against spinal cord injury (36). However, in a 2022 study, it was shown that there were no differences in lesion volume, glial cell reactivity, or functional recovery between female and male SD rats after SCI (33). Funding agencies such as the NIH have suggested that including both genders in studies can improve the translatability of preclinical findings to the human condition. However, there are significant challenges; two sexes would greatly increase the cost and complexity of the study, and the estrous cycle of female animals would also have an impact on the experimental results, increasing the variability of the results (32).

### 3.2 Selection of the injured segment

Most studied models were injured mainly at the thoracic spine level and were concentrated around T10 (7). The second most common injury site is the cervical level, which has some clinical relevance. Spinal cord injuries in humans usually occur more frequently at the cervical level and have more severe consequences, producing significant signs of neurological damage (1, 37). Spinal cord injuries are less common at the lumbar level (38).

Ethical requirements should be followed when selecting the segment to be injured. Lower levels of SCI stages, such as thoracic spinal cord injuries, should be selected while meeting scientific objectives. Injuries at the cervical level should be avoided, as they may be extremely painful for the animal and increase the difficulty of care as well as postoperative mortality (21, 39).

### 3.3 Selection of SCI damage level

SCI research focuses on two main aspects: first, to investigate the pathophysiologic processes and anatomical changes of SCI, and second, to search for and study potential repair pathways. When conducting SCI studies, it is crucial to select an appropriate injury model and control the degree of injury, the ceiling effect (i.e., improvement is maximized and further enhancement is difficult) and floor effect (i.e., the injury is too severe and it is difficult to observe the therapeutic effect) need to be avoided (40, 41). An inappropriate level of impairment can affect the accuracy of study results and follow-up. For example, when studying the effect of a drug on the repair effect of SCI, if the degree of injury is too light, rodents may recover quickly under their recovery ability, which will result in the purpose of the experimental design not being realized. Conversely, if the degree of injury is too severe, the high mortality rate of the animals may make it challenging to observe the effect of the drug treatment, leading to a statistically non-significant difference in results.

Given the differences in equipment and technology levels between laboratories and the unique characteristics of SCI models, researchers who lack experience in SCI modeling should use articles by other researchers as an initial reference and conduct preexperiments before formal experiments to determine the appropriate level of injury. Pre-experimentation not only helps researchers to be familiar with the experimental operation but also adjusts and optimizes the experimental parameters to ensure the stability and consistency of the damage model, which lays a solid foundation for the formal experiment.

Through pre-experimentation, researchers can assess the effects of different injury levels on animals’ behavioral, physiological, and pathological changes and the effectiveness of treatments. In addition, pre-experimentation also helps to identify potential problems and challenges, enabling researchers to take corresponding improvement measures in formal experiments.

## 4 Clamp-type SCI model modeling methods

### 4.1 Animal selection

Twelve-week-old SD female rats were procured from the Laboratory Animal Center of Southern Medical University. The mice were maintained under standard laboratory conditions, including a temperature of 22°C and 12 h of daylight per day. These animals had access to food and water at will. All animal experiments were conducted by the Guide for the Care and Use of Laboratory Animals and ethically approved by the Laboratory Animal Ethics Committee of Zhujiang Hospital of Southern Medical University (LAEC-2023-006).

### 4.2 Preoperative preparation

#### 4.2.1 Material and equipment

Gas anesthesia machine, operating microscope, thermostatic pads for animals, shaver, Skull grinding drills, surgical instruments (ophthalmic scissors curved round, pointed, toothed forceps, small curved forceps, proppers, scalpel blades, handles, ultra-thin hemostatic forceps, needle holders, curved discs, triangular needles, circular needles), forceps for molding, cotton swabs, gauze, iodine vapors, cavity towels, 2.5 mL syringes, and sutures. Saline, penicillin, isoflurane, gentamicin.

#### 4.2.2 Animal preparation

SCI surgery is a significant traumatic event for healthy animals. The ethical guidelines for animal experimentation should be strictly followed during the experiment to minimise the impact on animals. Preoperative rats do not require fasting, which can lead to the occurrence of hypoglycemia and increase the stress response of rats to SCI (42). So-called intraoperative vomiting and other conditions are unsubstantiated and need not be of undue concern (43). If prophylactic analgesia is deemed necessary, non-steroidal anti-inflammatory drugs (NSAIDs) or opioids (e.g., paracetamol and tramadol) can be employed preoperatively to enhance the survival rate of the rats. These agents can also be utilized for a brief period postoperatively (21, 44). In our experiments, we observe the postoperative status of rats and find that they can tolerate the surgery. We will, therefore, administer analgesic drugs before and 3 days after the operation. Although the use of analgesics interferes with some of our evaluation indicators, we should conduct animal experiments following ethical regulations, dialectically consider the effects of drugs on the experiments, and adjust the experiments to take into account factors such as the effects of drugs (45). Some experts believe using analgesics is necessary and reasonable and does not affect the functional aspects of the animal model (44).

For rats that have recently been transported or have undergone a significant change in their long-term living environment, at least 1 week should be allowed to adapt to the new environment to comply with ethical standards and prevent stress-related reactions from influencing the reliability of experimental results. Some studies have demonstrated that immediately following transport, mice exhibit notable alterations in behaviors such as rearing, climbing, grooming, feeding, and sexual behavior, and blood corticosterone levels increase threefold (46–49).

### 4.3 Anesthesia

Various anesthesia options are available, and isoflurane is considered a preferred gaseous anesthetic because of its safety and Controllability. Isoflurane allows the operator to adjust the depth of anesthesia easily, and the animal can rapidly awaken from anesthesia, which is particularly important in emergencies (50). It is believed that isoflurane has some neuroprotective effects in adult rats and toxic effects in young or aged rats (51). Other anesthetics for injection have a limited duration of anesthesia for a single injection of anesthetics and are more dangerous if additional is administered again, and the depth of anesthesia is insufficient. For anesthesia in rats, the concentration of isoflurane is usually set at 3% during the induction phase and reduced to 2% during the maintenance anesthesia phase.

### 4.4 Localization of the injury

**Figure 2:**
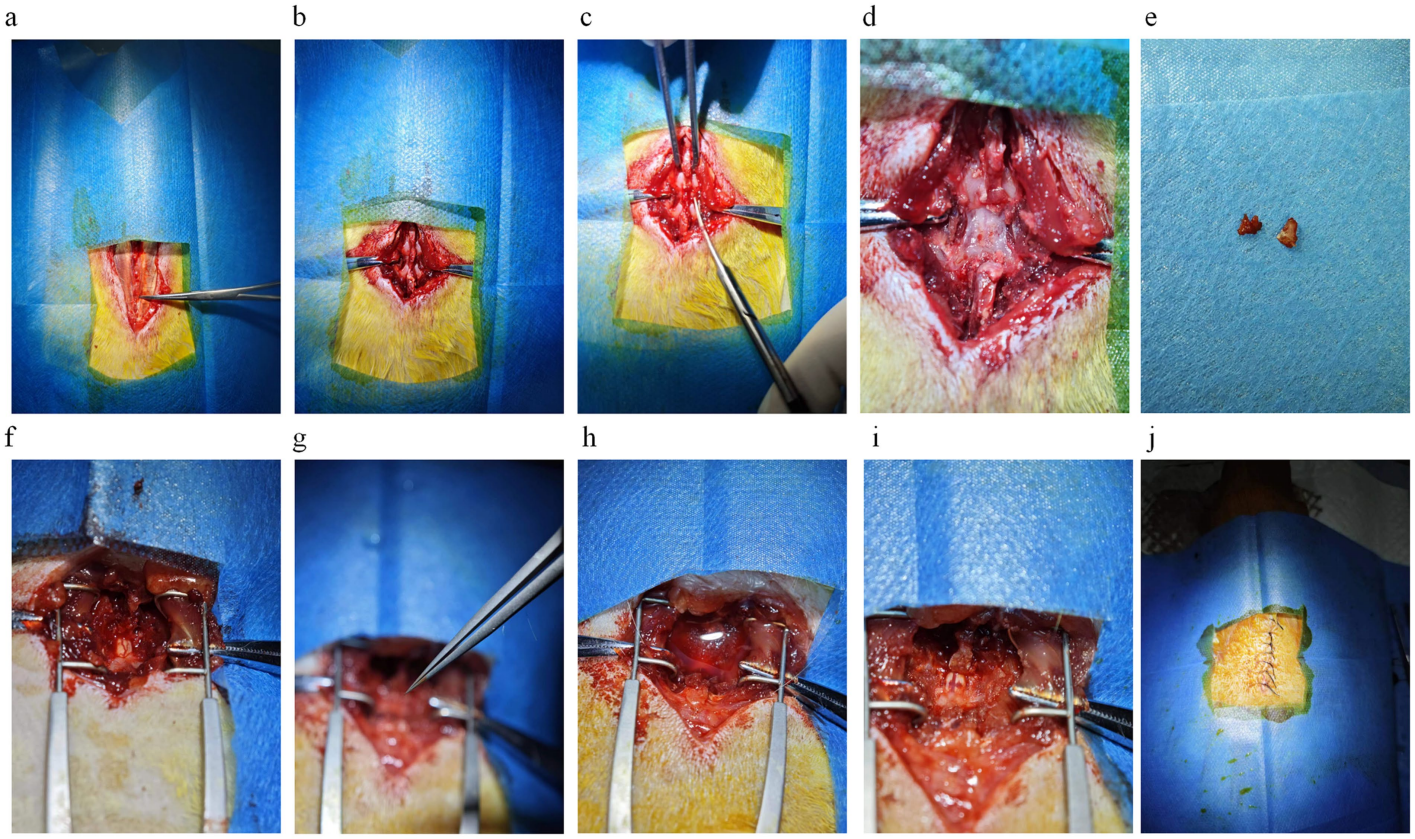
Key steps in spinal cord injury modeling. **(a)** The skin is incised to expose the tendon membrane on the muscle surface, which is attached to the spinous process on both sides. The place where the tendon membrane disappears from the spinous process when viewed from above is T10. The first place where the tendon membrane attaches to the spinous process is L1. The titanium alloy ultra-thin curved forceps in the figure point to the thoracic vertebral segment of T10, where the tendon membrane disappears from the spinous process. **(b)** Blunt separation of muscle tissue and exposure of vertebrae except T9/10/11. **(c)** The T9 spinous process is elevated, and the T10 spinous process is subtracted using dentate forceps. **(d)** The vertebral body is thinned with a mill/drill/cranial drill to visualize the blood vessels on the surface of the spinal cord tissue. **(e)** The vertebral body is removed with curved forceps. **(f)** The exposed spinal cord tissue is well vascularized and undamaged. **(g)** Special forceps clamp the spinal cord tissue for 20 s. **(h)** A large amount of blood flowed from the field of view after spinal cord injury and saline was used for irrigation and soaking. **(i)** Spinal cord tissue after spinal cord injury. **(j)** Intermittent suturing of skin tissue.

Following the administration of anesthesia and the transfer of the rats to a maintenance state, they were placed on a thermostatic pad to prevent a decline in body temperature due to intraoperative blood loss and anesthesia, which could result in death. Once the bony landmarks on the body surface had been localized, a suitable area for disinfection was selected. The local hair was wetted, and the skin was prepared using a shaver. After the preparation, the skin was again disinfected using iodophor cotton balls. The fingers of both hands were tensed against the skin, and a 3-cm incision was made at the designated site using a scalpel. Blunt separation of the subcutaneous tissue reveals the white sheaths on the muscles. Some studies have suggested that the location of the beginning of the first white muscle sheath is T10 (Figure 2a). However, in our group’s observation, this anatomical landmark is highly variable and can only be used as an auxiliary judgment indicator.

**Figure 3:**
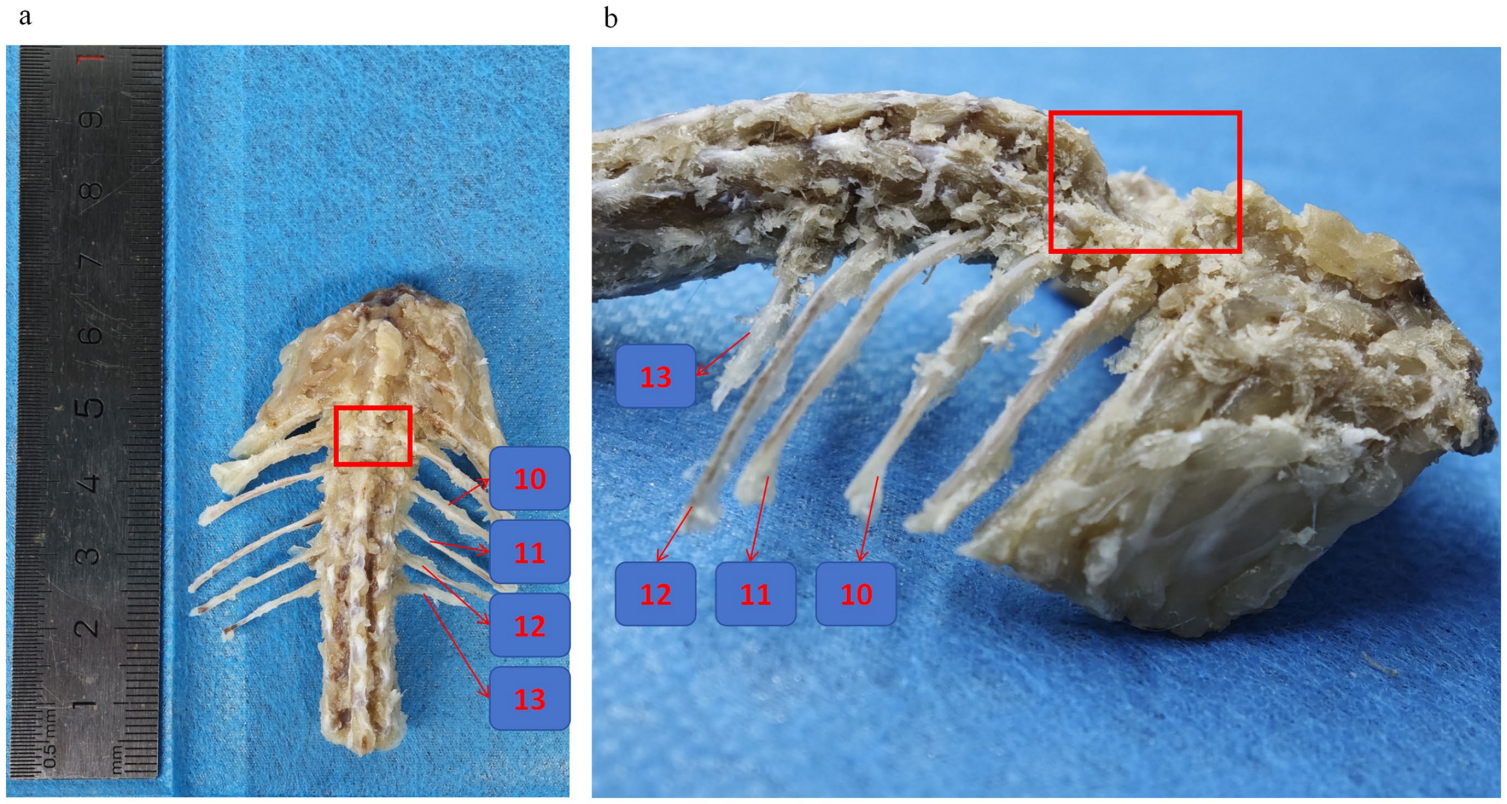
Rat thoracic and spinal skeleton. **(a,b)** Frontal view of spinal specimens from SCI rats with preserved ribs, the numbers represent the 13th, 12th, 11th, and 10th ribs, respectively, and the location of the red box is the T10 spinal segment where the vertebral plate was excised for SCI.

For the localization of T10, we propose the following two points: first, by palpation, determine the “high-low-high” pattern of the spinous processes of the three thoracic vertebrae, T9, T10, and T11. The notch is the T10 vertebra. The second localization method is to count the ribs, beginning with the floating ribs. However, it should be noted that the floating ribs are cartilage and require attention to the feel. Additionally, it is more challenging to identify the ready position of the floating ribs in mice. In rats, there are 13 vertebrae in the thoracic spine. The T10 vertebra can be identified by counting backward and upwards by three before retracing our steps to the thoracic spine (52) (Figure 3). It is recommended that novice researchers performing SCI modeling engage in preexperimentation. Once the localization principles have been applied, the rats are euthanized, and the intact vertebrae and ribs are removed. The isolated specimens are then utilized to verify the researcher’s feel and localization accuracy. This method increases the success rate of the surgery, reduces the damage to the rats, and ensures the experiment’s validity. Furthermore, it is a responsible practice for experimental animals and meets the requirements of experimental ethics and animal welfare.

### 4.5 Paravertebral muscle stripping

Muscle stripping represents a crucial step in the surgical treatment of SCI. The fundamental objective of this procedure is to employ a blunt separation technique to minimise damage to the muscle and surrounding tissues. During the muscle stripping of the spinal ramus, it is recommended to minimise the extent of muscle stripping while ensuring the feasibility of the surgical operation. This approach helps maintain a consistent degree of muscle stripping in rats during each operation, reducing variability and mitigating the effects on rat behavioral science (53). Some of the rats with excessive muscle stripping exhibited severe spinal deformity at the time of the final retrieval, which is consistent with the theory (54). Muscle stripping should be combined with a spatula in addition to blunt dissection, or the use of a scalpel or ophthalmic scissors can be used in place of a spatula; the key is to adapt the tool to the bone surface for stripping to reduce bleeding and aid in precise identification of bony structures (Figure 2b).

### 4.6 Removal of the vertebral plate

Laminectomy is a critical and challenging step in the process of establishing a SCI model, and the difficulty of its operation lies in the fact that the laminectomy must be performed without damaging the spinal cord tissues and with as little disturbance to the spinal cord tissues as possible (55, 56). Performing a laminectomy in the SCI model is an essential source of stimulation for the experimental animals, which can elicit varying degrees of physical response. For this step, we tried two approaches, the first being the nibbling method (55). After releasing the muscles on the surface of the vertebrae, the spinous process of T10 is cut off with scissors (Figure 2c). Then the spinous process of T9 is lifted with forceps, thus lifting the T10 vertebrae in the same direction, leaving some space between the spinal cord and the vertebral bone and reducing the risk of SCI when using the bur. The vertebral body plate is carefully thinned with the bur, taking care not to cut through the vertebral body plate, with the goal of turning the plate from white to a whitish-red color with the spinal cord vaguely visible (Figure 2d). An incision is then carefully made with ophthalmic scissors, and the vertebral plate is bitten through the breach with ultra-thin titanium curved forceps, and the remaining tissue is bitten off with regularly curved forceps after sufficient space has been bitten open (Figures 2e,f). During laminectomy, care must be taken to protect the spinal cord, and if necessary, the spinal cord must be blocked with a nerve stripper to perform the maneuver. The second method of laminectomy: For examiners familiar with the anatomy, the intervertebral space can be exposed by directly elevating the spinous process of T9 with forceps or clamping the spinous process of T10. The entire vertebral plate is removed using ophthalmic scissors (curved, rounded tip); this method is faster and more suitable for experienced examiners. There is no need to over-clean the two lateral walls of the spinal canal when removing the vertebral plate tissue because our forceps are finer and have enough room to maneuver. However, in the case of aneurysm clips, it is necessary to dissect the lateral structures on both sides of the spinal canal to place the clips, which may also have side effects such as bleeding.

### 4.7 Hemostatic

When performing SCI model surgery, the laminectomy is a high risk for bleeding. Saline is recommended for immersion and irrigation to control bleeding and effectively keep the surgical field clear. This method quickly removes blood from the field of view and has a hemostatic effect (Figure 2h).

In addition to saline, other hemostatic measures may be used, such as plain cotton balls, absorbent gelatin sponges, hemostatic gauze, or electrocoagulation. However, it is essential to note that some hemostatic materials may cause additional complications in SCI modeling, so care should be taken when selecting hemostatic materials (57). Before closing the surgical incision, it is essential to check for active bleeding sites to ensure complete hemostasis carefully. Considering that rats’ total body blood volume is only about 55 mL, bleeding of more than 10% can be life-threatening. Therefore, it is recommended that body fluids that may have been lost during the procedure be replenished by intraperitoneal injection of 2 to 3 mL of normal saline after the surgery (43). In addition, the following details should be noted: When using electrocoagulation for hemostasis, the intensity and time of electrocoagulation should be controlled to avoid thermal damage to spinal cord tissues. When using hemostatic materials, pressure on the spinal cord and surrounding tissues should be minimized to avoid interfering with the recovery of spinal cord function. During surgery, the physiological status of the rats, such as heart rate, respiration, and blood pressure, should be closely monitored to detect and treat potential complications promptly. Appropriate analgesic measures should be provided after surgery to reduce the pain and stress response of the rats.

By comprehensively considering these factors and taking appropriate preventive and treatment measures, the risk of bleeding during surgery can be minimized, and the success rate of surgery can be improved. At the same time, the rats’ postoperative recovery and the experiment’s scientific validity can be ensured.

### 4.8 Spinal cord injuries

After complete exposure of the spinal cord, the forceps used for specialized spinal cord injuries were removed, and the finger grip was positioned at the limiter position, ensuring that the long axis of the forceps was perpendicular to the long axis of the spinal cord. The forceps were then slowly closed, timing began immediately upon reaching the limiter position, and the forceps were released after 20 s (Figure 2g). Spinal cord injuries are usually accompanied by severe bleeding, which can be stopped entirely after rinsing and soaking with saline, and dark red ecchymosis can be clearly seen on the surface of the spinal cord tissue (Figure 2i).

### 4.9 Post SCI suture

After SCI surgery, the incision can be sutured once hemostasis is confirmed. During the suturing process, the muscle layer close to the spinal cord should not be sutured too tightly so that chronic compression caused by postoperative bleeding does not affect the recovery of the spinal cord tissues, which will change the consistency of the SCI. The interrupted suture technique should be used for suturing the outermost skin layer (Figure 2j). The purpose of this is to prevent the rats from removing the sutures themselves after surgery, as a continuously sutured wound that is removed may increase the risk of wound infection or even death. This careful suturing can optimize the postoperative recovery process and reduce the incidence of complications, thereby improving the accuracy of the experiment and the welfare of the rats.

### 4.10 Postoperative care

In SCI animal models, the cause of death is usually related to infection, but infections directly caused by surgery are rare due to strict adherence to aseptic principles during surgery. The urinary system is a high-prevalence area for infection, especially in the case of urinary retention that occurs after SCI, and urine retention in the urinary tract is prone to trigger infection. In addition, because the rats were paralyzed in both lower limbs after SCI and could only crawl, the friction and direct contact between the urinary system and the external floor or bedding also increased the risk of infection. Therefore, for rats undergoing SCI modeling, their mobility should be taken into account, and food should be reasonably placed to ensure that it is easily accessible to avoid lack of food due to improper placement of food, which may affect health recovery or even lead to starvation death. Bedding should be changed at least every 2 days, and the environment should be kept dry to reduce the risk of infection. The thermoregulatory ability of rats may be impaired after SCI (58), so it is necessary to maintain the environment at an appropriate temperature to avoid overcooling or overheating. Thermal insulation measures such as heat lamps or heating pads should be used if necessary. In general, postoperative exposure to infrared light is recommended to help SCI rats emerge from anesthesia. In addition, rats may experience pain after SCI, so the pain level should be assessed, and appropriate analgesics should be provided to improve their quality of life.

## 5 Modeling SCI complications and management

### 5.1 Manifestations and management of infection

Infection may manifest When rats are given assistance to urinate; the urine is cloudy in color with a peculiar odor. In severe cases, the urethral orifice is whitish and blistered, and the rat’s weight does not increase and decreases sharply, with decreased mobility, slow reaction, and poor mental state. The position of the abdomen touching the ground makes it easy to shed hair, and there are bedsores, which can be treated by applying erythromycin ointment. Observation of the hair condition of rats may also reflect their recovery: rats with good recovery have neat and clean hair, while rats with poor recovery may have messy, shedding, and unclean hair (43). To reduce the mortality rate due to urinary tract infection, we prevented it by intraperitoneal injection of penicillin after surgery and continued it until the rats regained their bladder reflexes and were able to urinate on their own. In case of urinary tract infection, we clean the perineum daily with iodophor and increase the frequency of assisted urination. As soon as signs of infection are detected, upgrading the antibiotic therapy by changing the regular penicillin to a second-generation cephalosporin antibiotic is recommended.

### 5.2 Bladder functional status analysis and management

After thoracic SCI, bladder function will recover spontaneously in most rats within 2 weeks. If there is still no recovery after 2 weeks, an appropriate extension of the observation period may be considered, but the maximum duration should not exceed 3 weeks. If there is still no improvement in bladder function, humane termination of the study is recommended.

**Figure 4:**
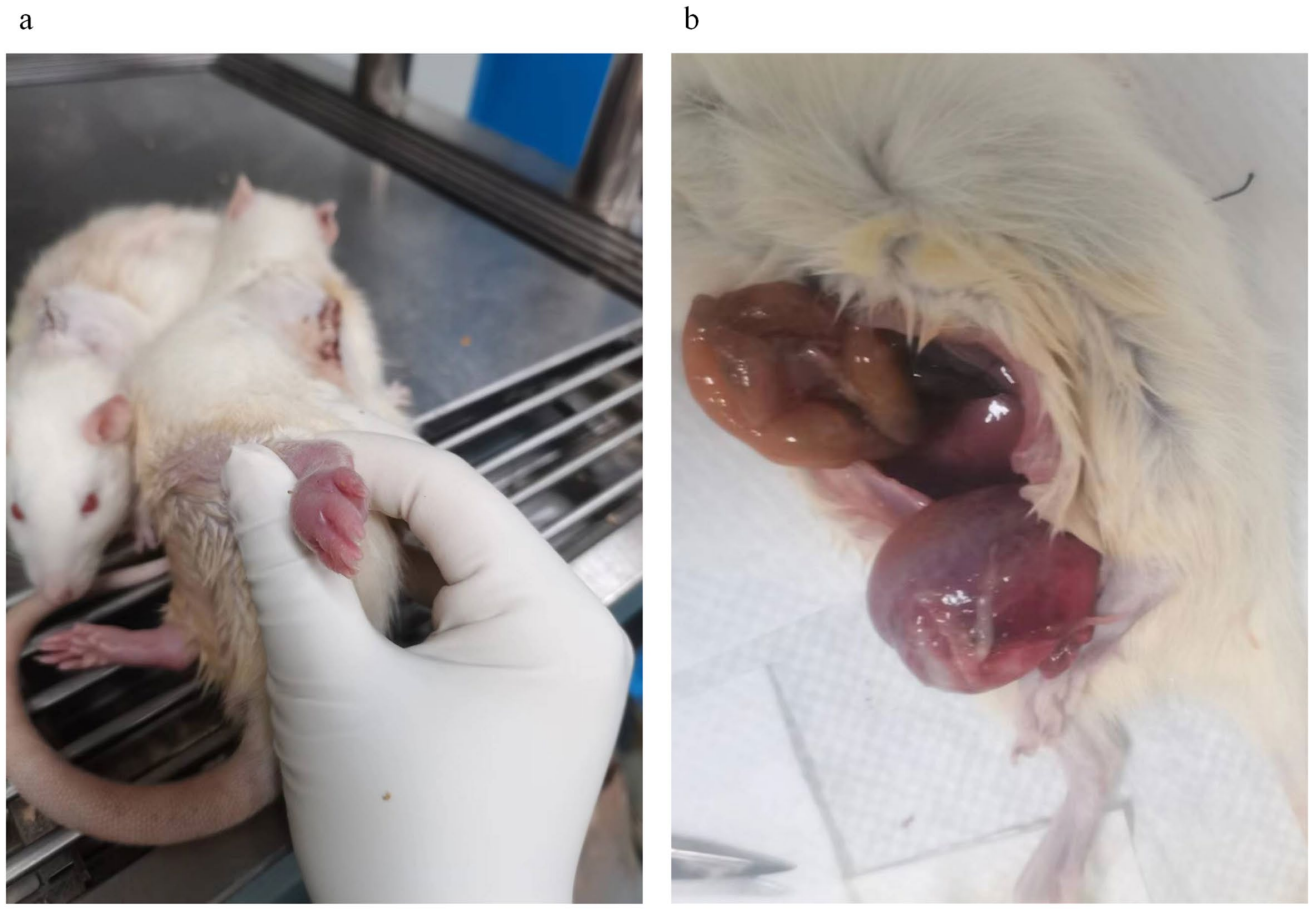
Common complications after spinal cord injury in rats. **(a)** Edematous rat feet days after spinal cord injury; **(b)** Neurogenic hematuria after spinal cord injury, enlarged bladder in rats killed by urinary retention.

If hematuria occurs during voiding assistance after SCI, it may be caused by a neurogenic bladder (59–61). At this point, the bleeding should be closely monitored. Blood volume is usually assessed by the color of the rat’s lips and eyes. If the color of the lips and eyes is red, fluid replenishment by intraperitoneal injection can be given appropriately. If the eye color is pale and activity is decreased, experience suggests that the rat may not survive more than 4 days. If further observation is required, the rat should be kept in isolation to avoid adverse effects on healthy rats in the same cage, such as removing sutures or tearing of incision skin, which would severely violate animal welfare principles. If the bladder is highly distended but unable to pass urine by any method of voiding, the outlet may be blocked by a blood clot formed by bleeding within the bladder. In this case, the bladder is hard to the touch, similar to nasal cartilage (Figure 4b). If the situation persists for a day without improvement, euthanasia should be considered.

### 5.3 Management of edema occurrence

If bladder function is not restored, urine retention in the bladder can easily damage renal function, resulting in decreased glomerular filtration rate and lower limb edema due to fluid retention (62–64). The mechanism of their lower limb edema may also be caused by long-term immobilization and paralysis of SCI (65). If bladder function is not restored, the edema is challenging to resolve independently. The phenomenon usually occurred first in the paw of one of the hind legs of rats (Figure 4a) and then involved both hind legs and, in severe cases, even the forelimbs. According to the experience of our research group, appropriate control of the amount of water consumed by the rats or short-term prohibition of water intake can alleviate this symptom. Replacement with hypertonic saline (30% NaCl) had a similar effect. However, these measures are used only for symptomatic management and are not recommended for long-term use. We also tried using injections of the diuretic furosemide but found that it was ineffective in reducing edema.

### 5.4 Humanized endpoint

Rats require timely termination for euthanasia performance or humane endpoints (21): SCI surgery is highly traumatic for rats, and especially postoperative infection control is a significant hurdle. In order to meet ethical requirements and not cause undue suffering to the animals, we propose a humane endpoint. We recommend that rats be euthanized under the following conditions:

1. Rats that have lost more than 20% of their body weight and show no trend toward recovery.
2. Rats that have not recovered bladder function for over 2 weeks. Rats with enlarged bladder with a hard texture and suspected blood clot blockage in the bladder with no improvement after 2 days of observation.
3. Lethargy and apathy, rats with little or no response to external stimuli.
4. Self-mutilation: rats with two or more phalanges missing from the limbs.

Two or more of the above conditions may occur in the same rat, but in any one of these cases, we recommend euthanasia, execution with CO2, or tissue collection or sampling.

## 6 SCI model specimen collection

### 6.1 Spinal cord sample collection

Spinal cord tissue is very peculiar, and soon after the death of the rat, the spinal tissue liquefies and becomes difficult to remove, or the removed sample is basically out of shape. Depending on the purpose for which the spinal cord tissue is removed, we use different fluids for perfusion. Paraformaldehyde is recommended for perfusion in morphological experiments such as HE staining, immunohistochemistry, or immunofluorescence. In rats perfused with paraformaldehyde, the spinal cord tissue remains liquefied for a longer period, allowing the investigator a longer period to remove the tissue. If the spinal cord tissue is subsequently used for molecular biological tests such as WB, we recommend using PBS or saline for perfusion and changing the commonly used method of collecting the tissue on ice to collecting the tissue on dry ice to prevent the tissue from liquefying too quickly. It is difficult to remove the spinal cord tissue intact before it liquefies, unlike mice whose bone tissue is thin and can be easily removed from the vertebral body with ophthalmic scissors. Rats require the use of curved forceps to nibble away, using scissors to make an incision below or above the injury site, and curved forceps to remove the vertebral bone protecting the spinal cord tissue in the direction of the notch. After the spinal cord tissue has been completely removed from the injury site, the end of the spinal cord is lifted with toothed ophthalmic forceps, and the nerve roots involved are carefully cut with ophthalmic scissors. The spinal cord tissue at the injury site especially has a high probability of scarring and the presence of strong adhesions, which should not be pulled and pulled but should be freed from obstacles one by one with ophthalmic scissors. After the spinal cord tissue is removed, the spinal cord tissue is immediately placed on a prepared paper sheet, which is then placed with the spinal cord tissue in paraformaldehyde for fixation or in a freezing tube and then stored in liquid nitrogen. The paper sheet is extremely useful for labeling the head and tail ends of the spinal cord, as well as facilitating characterization of the soft spinal cord tissue so that it is not fixed in an odd shape that would interfere with subsequent surgery.

### 6.2 Obtaining rat blood

**Figure 5:**
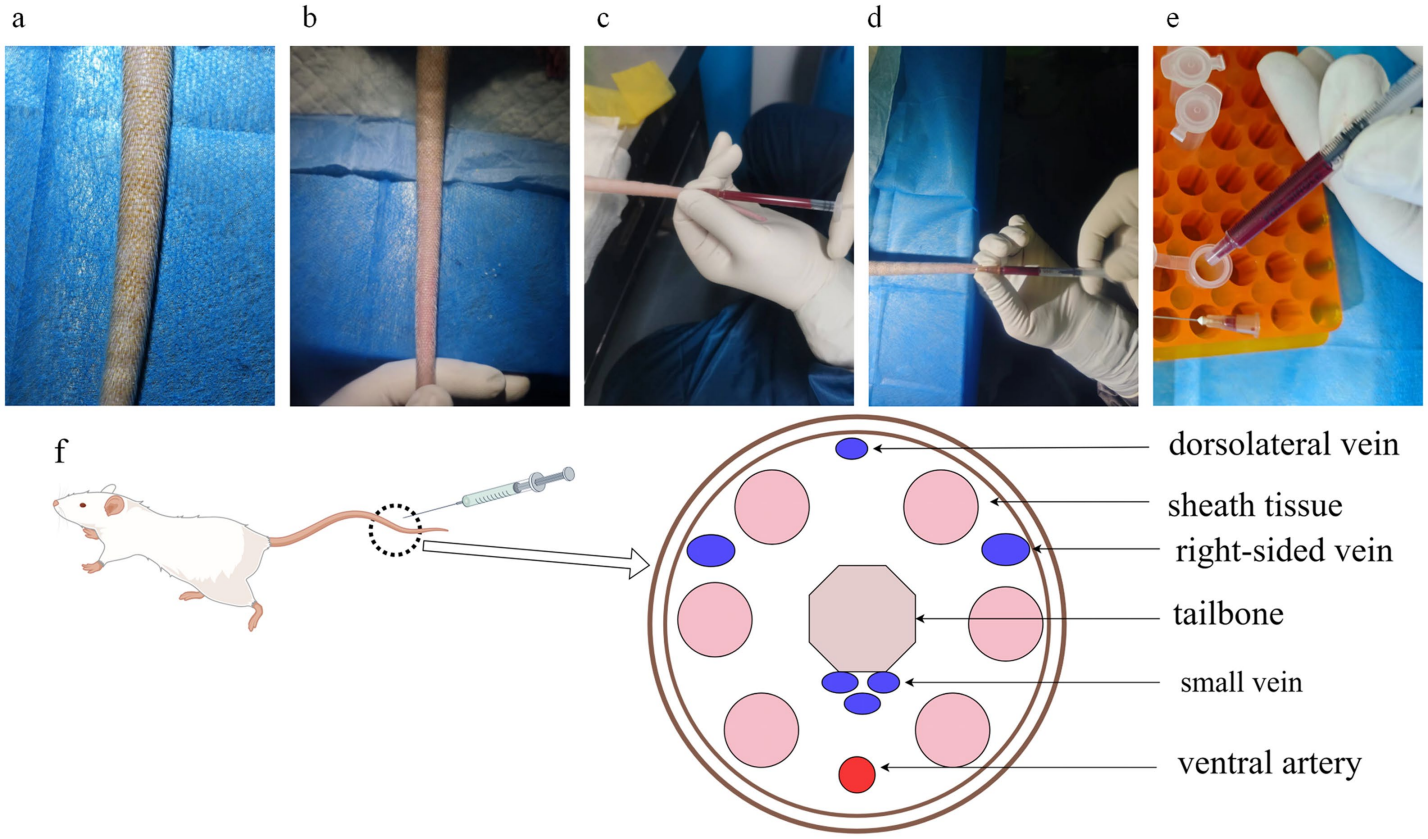
Rat tail vein blood sampling and tail anatomy. **(a)** The tails of elderly rats are dirty, and the skin surface is scaly, making it inconvenient to enter the needle for direct tail vein blood sampling; **(b)** The tail veins of rats soaked in warm water and scrubbed with alcohol are complete and clear, which makes them suitable to enter the needle; **(c)** Side view of blood sampling posture; **(d)** Front view of blood sampling posture. The posture can fix the needle tip in the tail vein, and it is convenient to adjust the needle tip’s direction and depth of the needle; **(e)** When transferring the blood from the syringe, remove the needle to prevent hemolysis caused by destroying the red blood cells with high-pressure when transferring blood; **(f)** Cross-sectional anatomical drawing of a rat tail, with veins in blue and arteries in red, and veins on both sides of the tail are relatively thicker, which is suitable for drawing blood.

Most researchers may need to collect blood samples to measure some biological indicators, and there are several ways to collect blood, including blood collection from the retro-orbital vein, blood collection from the external jugular vein, blood collection from the rat heart, blood collection from the rat tail vein, and blood collection from the abdominal aorta. More options are available if blood is collected during final sampling, and the technical requirements are less demanding. For studies requiring continuous monitoring of the blood sample or continued survival of the animal after blood sampling, the only appropriate methods are tail vein and external jugular vein blood sampling. The latter is demanding and requires highly trained technicians, whereas tail vein sampling is much easier. Tail vein blood collection requires the active cooperation of the rat, controlled tail twisting, and generally poor results due to the rat brake. By using isoflurane anesthesia, our research group has been able to shorten the procedure time and increase the success rate dramatically. Older rats are less capable of self-cleaning, have dirtier tails, and have formed keratinized scales on the tail skin, which are difficult to puncture (Figure 5a).

Specifically, the rat tail was first washed with hot water or soaked with warm water and then wiped with alcohol gauze to ensure tail hygiene and make the tail vein more apparent (Figure 5b), and blood was immediately collected using a 1 ML syringe (needle model 0.5*20 mm), taking care to collect blood from the distal end as far as possible. There are four tail veins in the rat, and the dorsal vein is relatively thin, so it is recommended that the thicker veins on both sides be selected for manipulation (Figure 5f). The closer to the tail end of the rat, the shallower the vein is, so it is easy to find the depth of the needle. However, the closer to the tail end, the smaller the tail vein’s diameter, making it more challenging to hit. Finally, we proposed to insert the needle in the posterior third of the rat tail (Figure 5c). After recognizing the location of the tail vein, the needle tip is angled upward, the needle is inserted into the skin of the tail, and the plunger of the syringe is pulled back a certain distance to create a negative pressure in the syringe. After the negative pressure is formed, the needle is inserted further toward the position of the tail vein. Due to the formation of negative pressure, the blood will be sucked into the syringe very quickly after piercing the blood vessel, and we can see the blood at the connection between the needle and the syringe. After seeing the blood, the tip of the needle has reached the depth of the blood vessel, and it is necessary to stop the needle at 30° immediately, reduce the angle between the needle and the blood vessel, and advance the needle as far as possible in a parallel direction. We can clearly see the needle tip advancing in the blood vessel, entering about 1.5 cm. Carefully change the position of the hand, holding the syringe and rat tail parallel between the thumb and forefinger of the left hand, and slowly pull the plunger with the right hand (Figure 5d). While pumping and pulling the plunger, observe whether the blood inside the syringe slowly increases, otherwise moderately withdraw the needle tip parallel to the needle, or adjust the angle of needle entry, and the blood inside the syringe continues to increase, indicating that the adjustment is successful. Because of the need to observe the blood in the syringe over time, the syringe and needle connection where the blood is drawn should be transparent. If successive adjustments still fail to draw blood successfully, it is recommended that the syringe be withdrawn, the eye of the needle be pressed with a cotton ball to stop bleeding, and then the proximal end of the eye of the needle be selected for another blood draw or replaced with another caudal vein.

When transferring blood to the EP tube, the needle was removed to prevent blood from being pumped out of the needle at high speed and pressure, which would cause hemolysis and affect the quality of the blood sample (Figure 5e). Blood samples were allowed to stand at room temperature for 30 min to allow clotting and then centrifuged at 2500 g for 20 min. The serum was separated from each sample and stored at −80°C.

## 7 Post SCI evaluation

Spinal cord injuries are evaluated in many ways and, according to current research advances, are mainly evaluated through behavioral tests, pathology, biology, neurophysiology, imaging, musculoskeletal, cardiovascular, and respiratory. More than 50% of the articles chose both types (pathology + behavioral) to evaluate SCI outcomes (7).

### 7.1 Behavioral evaluation

More than half of the basic research articles on SCI will use behavioral testing to assess the state of recovery from SCI. Behavioral assessments can visualize the state of SCI recovery, which is consistent with our goal in the clinic to improve the function and quality of life of patients with SCI. Behavioral assessments can be broadly categorized into motor function assessments and sensory function assessments; the BBB score is widely used to assess hindlimb function in rats, while the BMS score is widely used to assess motor function in mice (7, 66). Sensory function assessment includes cold stimulation test, heat stimulation test, and Seamus. Von Frey or Semmes-Weinstein test, and the rest include grid crawl test, balance beam walking test, gait analysis, inclined plate test, and Tarlov method (67–71). Although there are many methods for sensory and functional assessment, according to the current state of research, researchers are more concerned about the recovery of motor function after SCI, and there is more movement assessment during behavioral analysis. However, behavioral experiments have significant challenges, including high variability of results and multiple confounding factors, which require larger sample sizes to avoid false-positive or false-negative results. Common factors affecting behavioral experiments include the timing of the assessment (e.g., different times of the day), the mood of the assessor, and the order of the assessment, especially in those behavioral experiments that are highly subjective (22).

**Figure 6:**
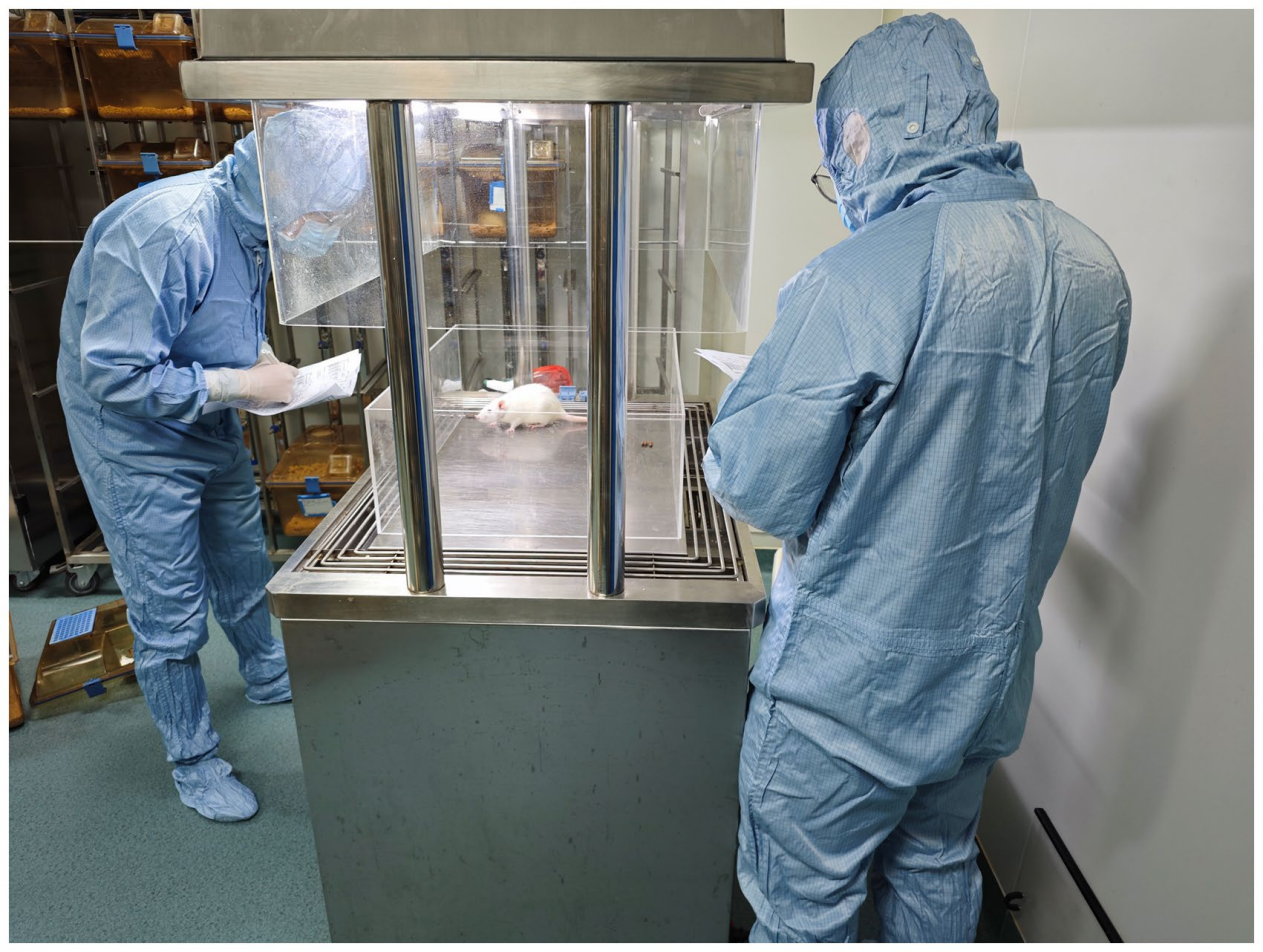
BBB scoring on-site. BBB scoring requires two people to be double-blind, with each person standing diagonally across the observation area, observing for 4 min, and taking notes according to the rules.

For example, the BBB score (Figure 6), divided into three levels of 21 points, is used primarily to assess hindlimb function in rats with mild and moderate SCI (72). The scorers need to be trained to ensure the accuracy of the scoring results, and the differences between the reaction speed and understanding of the scoring rules of novice scorers and experienced scorers are apparent (66). To solve the above problems, it is recommended to use video recording of behavioral experiments to facilitate reassessment. Second, we can increase the sample size of each group and combine subjective and objective behavioral experiments. Special attention should be paid to the fact that BBB scores cannot be treated as mere numbers, and the functional difference between scores 5 and 10 is different from the functional difference between scores 10 and 15 (67). It is not appropriate to consider the scores as normally distributed and analyze them using parametric statistics, but some experts believe that it has little effect on the results of the experiment, and many articles have been analyzed using parametric statistics (72).

### 7.2 Pathological assessment

Pathologic assessment of SCI recovery focuses on evaluating the extent of SCI tissue cavitation, scar tissue, myelin sheaths, specific nerve fiber bundles, neoangiogenesis, and inflammatory cell infiltration. Techniques such as immunohistochemistry and immunofluorescence can also be used to localize and quantify specific protein molecules and some tracing of nerve fiber bundles. Common stains include HE staining, which can be used to assess the size of the spinal cord tissue cavity (73); MASSON staining to assess the amount of scar tissue; LFB staining to assess myelin and observe nerve demyelination; and Nissl staining to observe the number of surviving neurons localized to the injury (74, 75). Immunohistochemistry or immunofluorescence is used to label the neovascular endothelial cell markers CD31 or CD34, and CD68 is used to label macrophages to observe inflammation (76–78). GFAP, Iba1, Olig2, and NeuN have been used to label astrocytes, microglia, oligodendrocytes, and neurons (79–81).

In addition to some of the stains mentioned above, nerve tract tracing has gradually been widely used in recent years (82, 83). Nerve tract-tracing techniques have been used for a long time, and their development can be roughly divided into four stages. First, there were the early traditional methods, such as Nissen staining and Nauta silver staining, which could label damaged nerve cells and fibers, but were limited to the analysis of fixed tissues and could not be used *in vivo*. Subsequently, the introduction of second-generation techniques such as horseradish peroxidase, cholera toxin *β*-subunit, and tetanus toxin allowed scientists to follow nerve fibers in living samples, further advancing the understanding of the dynamics of neural connectivity (83, 84). In the third generation, the use of chemical markers such as biotinylated dextran amine (BDA) and phytohaemagglutinin (PHA-L) provided more precise immunochemical reaction tools for detailed analysis of complex neural networks (85, 86). The latest fourth-generation tracer technologies use viral vectors, such as rabies virus and adeno-associated virus, for transfection to express fluorescent proteins within specific neuronal cells, allowing scientists to accurately track the dynamics of specific neural circuits in living animals (87, 88).

## 8 Conclusion

The quality establishment of a SCI animal model is the cornerstone of SCI research and one of the most critical factors in the experiment. When selecting a model, researchers must consider the Controllability of the model’s level of injury, ease of modeling, and fit with the research objectives. Although there are many known and unknown differences between rodents and human SCI, most researchers still prefer to use rodents to model SCI. Many rodent-based animal models of SCI have yielded promising results, and research is gradually moving toward using large mammalian models and clinical trials that more closely resemble human SCI.

Although there is a wide variety of SCI models, most of the modeling processes and steps are basically the same, except for the most critical differences in the injury process. For researchers new to the field, experience with the details of modeling, postoperative care, and management of common complications can significantly reduce mortality and intermodel variability in SCI models.

Scientific assessment after SCI requires and integrates multiple factors, and relying solely on behavioral scores, histological assessments, or molecular biological indicators (e.g., inflammatory factors, growth factors) often does not accurately reflect the pathophysiological processes and repair effects after SCI.
